# Finite-Time Thermodynamic Model for Evaluating Heat Engines in Ocean Thermal Energy Conversion

**DOI:** 10.3390/e22020211

**Published:** 2020-02-13

**Authors:** Takeshi Yasunaga, Yasuyuki Ikegami

**Affiliations:** Institute of Ocean Energy, Saga University, 1 Honjo-Machi, Saga 840-8502, Japan; ikegami@ioes.saga-u.ac.jp

**Keywords:** finite-time thermodynamics, reversible heat engine, normalized thermal efficiency, maximum work, exergy, entropy generation

## Abstract

Ocean thermal energy conversion (OTEC) converts the thermal energy stored in the ocean temperature difference between warm surface seawater and cold deep seawater into electricity. The necessary temperature difference to drive OTEC heat engines is only 15–25 K, which will theoretically be of low thermal efficiency. Research has been conducted to propose unique systems that can increase the thermal efficiency. This thermal efficiency is generally applied for the system performance metric, and researchers have focused on using the higher available temperature difference of heat engines to improve this efficiency without considering the finite flow rate and sensible heat of seawater. In this study, our model shows a new concept of thermodynamics for OTEC. The first step is to define the transferable thermal energy in the OTEC as the equilibrium state and the dead state instead of the atmospheric condition. Second, the model shows the available maximum work, the new concept of exergy, by minimizing the entropy generation while considering external heat loss. The maximum thermal energy and exergy allow the normalization of the first and second laws of thermal efficiencies. These evaluation methods can be applied to optimized OTEC systems and their effectiveness is confirmed.

## 1. Introduction

The Earth absorbs approximately 47% of solar energy as heat. About 71% of the Earth’s surface is covered by ocean. The ocean’s thermal energy is the temperature difference between the surface and its deeper zones. Ocean thermal energy conversion (OTEC) has significant potential worldwide and can enroll a stable baseload because the ocean has abundant heat stored from the sun and the cold seawater in the ocean’s deep zone remains constant throughout the year. Considering the functions that contribute to the stable energy supply in the grid, islands in tropical areas can be some of the best practical sites. Therefore, the temperature variation over time is slower than the fluctuation of other renewable sources, such as wind, solar power, ocean waves, and currents. In addition, discharged seawater can be applied in other industries. For example, the thermal energy after OTEC use can be used to desalinate seawater and deep seawater is applicable in aquaculture, agriculture, drinking water, cosmetics, and other industries and can have a very large economic impact [1]. 

In OTEC, the theoretical thermal efficiency is low due to the small temperature difference in heat sources, which is a maximum of approximately 25–30 K. This temperature difference between the two seawaters is the energy required to transfer the heat that drives a heat engine to produce power. In this study, both warm and cold seawater are defined as the heat source. As heat is transferred from seawater (heat source) to a heat engine, the seawater temperature varies and the effective available temperature difference in the heat engine—the typical temperature between evaporation and condensation for the working fluid in the heat engine—will decrease accordingly because of the sensible heat of seawater and the finite quantity heat source. Some studies have attempted to increase the effective temperature difference from the heat source using a non-azeotropic mixture as a working fluid, by staging to apply multiple temperature levels, or by using a heat engine for cascade utilization [2,3,4,5,6,7,8]. 

The magnitude of the work from a heat engine is the result of the balance in the thermal energy input into the heat engine and the efficiency depending on the effective temperature difference of the heat source. Wu identified the relationship between the work from a heat engine and the heat transfer performance of heat exchangers in OTEC in consideration of the finite-time process on the heat exchange in cases where the temperature change in the heat source is negligible while using a heat engine [9]. Ikegami and Bejan showed the theoretical thermal efficiency by considering both the OTEC system’s internal power consumption and the seawater pumping powers and maximized the first-law and second-law efficiency [10]. Wong-Yong and Sang-Soo theoretically analyzed the power output from Carnot and Lorentz cycles and showed that the power output in the Lorentz cycle will be twice that of the Carnot cycle from a finite temperature reservoir. These studies showed that a cycle’s thermal efficiency will be a function of the heat-source temperatures. Therefore, although the cycle’s thermal efficiency is applied to evaluate the OTEC system, the thermal efficiency no longer represents the system performance because the maximum thermal efficiency condition does not correspond to the heat engine’s maximum power output condition [11]. 

The consideration of a finite energy conversion system is dealt with in the field of finite-time thermodynamics (FTT) [12]. In FTT, Nokiv [13] and Curzon and Ahlborn [14] revealed the thermal efficiency at the maximum power output using the Carnot heat engine only employing the heat-source temperature. Bejan [15] modeled the entropy generation rate in a power plant using high and cold seawater flows and clarified the relationship between heat engines and the minimum entropy generation rates of heat sources at the power plant’s maximum power output. Ikegami and Bejan [10] considered the entropy generation of heat-source streams due to pressure drops caused by heat exchanges, and thereby derived the maximum power and thermal efficiency from the cycle. Gordon and Huleihil [16] and Gordon and Orlov [17] identified the chemical potential and effect of mass transfer on the infinite and finite reservoirs. Brogioli et al. [18] conducted a thermodynamic analysis on distillation for thermal regenerative salinity gradient for low-grade thermal energy. Chen et al. [19] summarized the cycles’ various thermal efficiencies at maximum power as modeled for additional or other constraint conditions. Many researchers have discussed and augmented proposals for the upper limit of a heat engine’s thermal efficiency at the maximum power output, both in the context of nuclear power plants and other energy conversion systems [12,20,21,22,23,24,25,26,27]. Wu first applied FTT to the OTEC system with the assumption of an infinite flow reservoir of seawater [9]. Their performance index was the ratio of the work output from the heat engine over the maximum available power as the second law efficiency. In the case of power extraction from limited single-phase streams, Bejan and Errera [28] maximized the available power from an irreversible heat engine by considering the heat exchanger’s length and performance; they used the ratio of available power over the idealized work output from the irreversible heat engine as their performance index. Ohman and Lundqvist [29,30] introduced the Fraction of Carnot cycle (FOC) as a performance indicator, which is the ratio of actual thermal efficiency over the integration of the Carnot thermal efficiency divided into n blocks, and Sinama et al. [11] introduced the same concept for OTEC evaluation. Morisaki and Ikegami [6,7,31] investigated multistage Carnot cycle performance and used the maximum power efficiency which is defined as the work per maximum work. Acikkalp [32] compared various performance indexes such as the ecological function criteria, maximum available work, and ecological coefficient of the performance assuming that the thermal energy vanishes at the ambient condition. Radcenco et al. [33] theoretically obtained the maximum–maximorum power and economical–ecological operation regimes as a function of thermal resistance and the temperature differences between heat sources and between the heat source and the Carnot heat engine and clarified the difference in the total entropy generation for both regimes. Yasunaga et al. proposed the concept of a new heat exchanger evaluation method that could consider the irreversibility of the energy conversion system and that could be applied to plate-type heat exchangers [34]. Kevin et al. compared the performance of OTEC based on the seawater side heat transfer performance and pressure drop [35].

To evaluate the system performance of a low-grade thermal energy conversion system (LTEC), Morisaki and Ikegami introduced the maxim power ratio, which is defined as the available power from a heat engine over the maximum power output using a Carnot cycle with an ideal heat exchanger [6]. Yasunaga and Ikegami [36] proposed a normalized thermal efficiency for LTEC that uses the available thermal energy stored in seawater as the sensible heat and as the input energy into the heat engine instead of the heat transfer rate. The normalized thermal energy will be effective in the energy conversion performance; however, the efficiency can decrease to below the conventional thermal efficiency due to small temperature differences and may mistakenly give the impression that the system has quite low performance. Therefore, evaluating the system practically requires the second law efficiency based on FTT. 

This study proposes an effective performance evaluation method for OTEC systems to evaluate the energy conversion performance from heat into work as the normalized thermal efficiency (the first law of thermodynamics) and the effectiveness of the system’s performance, which is defined as the exergy efficiency (the second law of thermodynamics) based on FTT for the productive design of the OTEC system, the practical use of the evaluation method, and to apply the exergy and, thus, calculate the theoretical potential energy of OTEC. 

## 2. First Law of Thermodynamics

### 2.1. Available Work with Ideal Conditions 

In conventional thermodynamics, the heat source for a heat engine is recognized as a higher temperature heat sink rather than the environmental condition, which generally uses the atmospheric temperature. Here, conventional power plants mainly use chemical reactions and air, water, or seawater is used as a coolant for a heat engine; therefore, the high-temperature heat source’s energy density is sufficiently larger than the cold source. However, with an OTEC, the two heat sources have equivalent energy density so evaluating their thermal efficiency requires deliberate attention.

**Figure 1:**
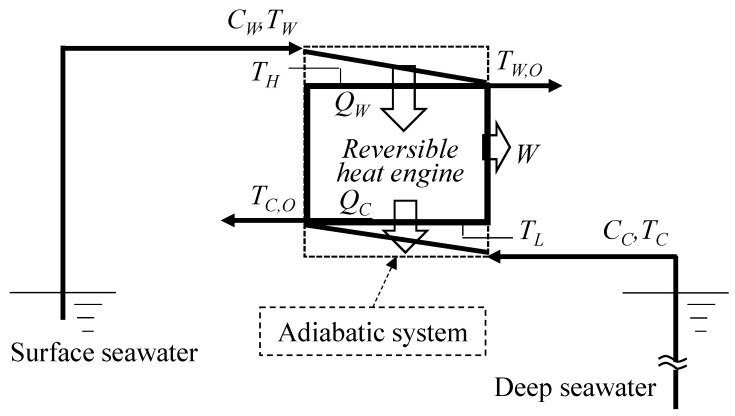
Concept of a reversible heat engine driven by high- and low-temperature streams.

**Figure 2:**
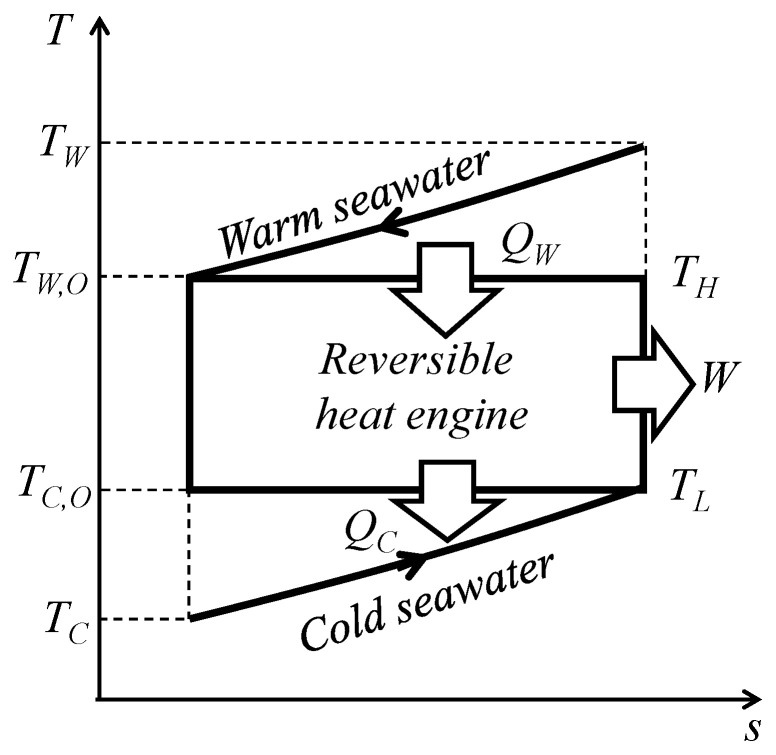
Conceptual *T*–*s* diagram of a reversible heat engine.

Figure 1 and Figure 2 are respectively the concept and conceptual *T*–*s* diagram of OTEC systems using a reversible heat engine. The reversible heat engine is driven by thermal energy stored as sensible heat with warm surface seawater and cold deep seawater. By means of heat exchange processes, the temperature of the warm seawater *T_W_* drops and that of the cold seawater *T_C_* rises. The heat exchange process is idealized and the outlet temperature of the seawaters *T_W_*_,*O*_ and *T_C_*_,*O*_ will reach the same temperature as the heat engine *T_H_* and *T_L_* with isobaric changes in the heat sources by ideal heat exchangers. The heat transfer rates and—according to the law of energy conservation—the work output of the heat engine *W* can be calculated using the following equations:

(1) $$Q_{W}={\left(mc_{p}\right)}_{W}\left(T_{W}-T_{W,O}\right)$$

(2) $$Q_{C}={\left(mc_{p}\right)}_{C}\left(T_{C,O}-T_{C}\right)$$

(3) $$W=Q_{W}-Q_{C}$$

where *Q* [kW] is the heat transfer rate, *m* [kg/s] is the mass flow rate, and *c_p_* (kJ/kgK) is the specific heat assumed constant in the temperature variation in the OTEC. The subscript terms *W*, *C*, *HS*, and *O* are the warm surface seawater, cold deep seawater, total heat source, and outlet, respectively. Here, the dependence of the specific heat on the temperature is assumed negligible since the temperature changes in seawater are sufficiently small. According to Figure 1 and Figure 2, the reversible heat engine received process is (1) isothermal expansion receiving the heat of *Q_W_* from warm seawater, (2) isenthalpic work output, (3) isothermal compression releasing the heat of *Q_C_* to cold seawater, and (4) isenthalpic compression. 

The heat engine’s thermal efficiency *η_th_* is defined as:

(4) $$\eta_{th}=\frac{W}{Q_{W}}$$

To focus on the maximum available work in the process, it is supposed that the process has ideal heat exchangers and an ideal (reversible) heat engine. For the ideal heat exchangers, it is assumed that the temperature of the heat engine after the heat exchange process corresponds to the outlet temperatures of the heat source (the pinch point temperature is zero):

(5) $$T_{H}=T_{W,O},\;T_{L}=T_{C,O}$$

In a reversible heat engine, the internal irreversibility is zero, i.e., no entropy is produced in the cycle; hence:

(6) $$\oint ds=\frac{Q_{W}}{T_{H}}-\frac{Q_{C}}{T_{L}}=\frac{Q_{W}}{T_{HO}}-\frac{Q_{C}}{T_{C,O}}=0$$

From Equations (1)–(3), (5) and (6), the work output of the heat engine will not be one degree of freedom from either *T_C_*_,*O*_ or *T_W_*_,*O*_. The warm seawater outlet temperature and the cold seawater outlet temperature can be respectively expressed as:

(7) $$T_{W,O}=\frac{{\left(mc_{p}\right)}_{W}T_{C,O}T_{W}}{{\left(mc_{p}\right)}_{C}\left(T_{C,O}-T_{C}\right)-{\left(mc_{p}\right)}_{W}T_{C,O}}$$

(8) $$T_{C,O}=\frac{{\left(mc_{p}\right)}_{C}T_{C}T_{W,O}}{{\left(mc_{p}\right)}_{C}T_{W,O}-{\left(mc_{p}\right)}_{W}\left(T_{W}-T_{W,O}\right)}$$

**Figure 3:**
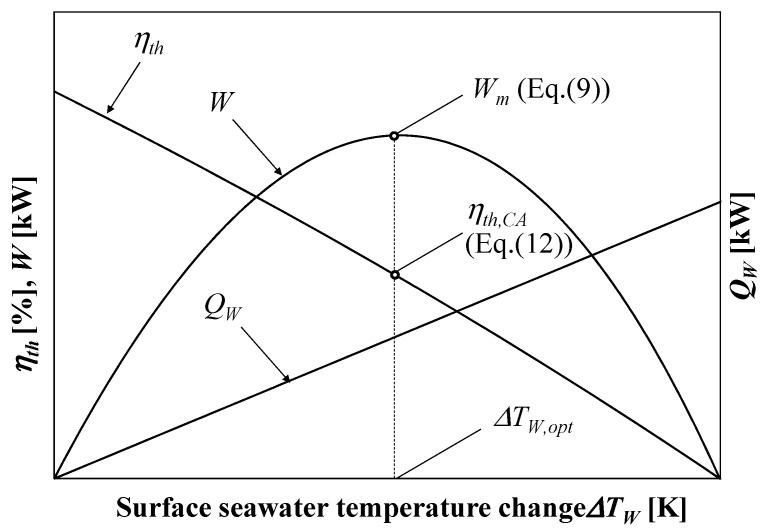
Concept of the relationship between the surface seawater temperature change and thermal efficiency *η_th_*, heat flow rate *Q_H_*, and power output *W*.

Figure 3 shows the thermal efficiency, work output and heat transfer rate of warm surface seawater as a function of the surface warm seawater temperature change. According to Figure 3, the thermal efficiency decreases monotonically with increases in the surface seawater temperature and the heat transfer rate of warm seawater increases in proportion to increases in the surface seawater temperature change. As a balance between the efficiency and heat transfer rate, the work shows a parabolic curve. Therefore, the optimum surface seawater change condition differs in terms of thermal efficiency and work output. The difference between the trends of thermal efficiency and the work shows that the thermal efficiency cannot be used to evaluate the heat engine’s performance.

Using Equations (1)–(3) and (7) or (8), if the work of a heat engine *W* is one degree of freedom from *T_W_*_,*O*_ or *T_C_*_,*O*_ and maximized as $\partial W/\partial T_{W,O}=0$ or $\partial W/\partial T_{C,O}=0$ then the maximum work *W_m_* can be derived as follows [36]:

(9) $$W_{m}=\frac{\;{\left(mc_{p}\right)}_{W}{\left(mc_{p}\right)}_{C}}{{\left(mc_{p}\right)}_{W}+{\left(mc_{p}\right)}_{C}}{\left(\sqrt{T_{W}}-\sqrt{T_{C}}\right)}^{2}$$

(10) $$T_{W,O,opt}={\left(mc_{p}\right)}_{W}T_{W}+{\left(mc_{p}\right)}_{C}\sqrt{T_{W}T_{C}}$$

(11) $$T_{W,O,opt}={\left(mc_{p}\right)}_{W}T_{W}+{\left(mc_{p}\right)}_{C}\sqrt{T_{W}T_{C}}$$

When the work is maximized, the thermal efficiency can be derived using Equations (1)–(4) and (9)–(11):

(12) $$\eta_{th,CA}=\frac{W_{m}}{Q_{W}}=1-\sqrt{\frac{T_{C}}{T_{W}}}$$

The efficiency Equation (12) is well-known as the Curzon–Ahlborn thermal efficiency [12,14]. According to Equation (9), *W_m_* will be maximized when ${\left(mc_{p}\right)}_{W}={\left(mc_{p}\right)}_{C}$, whereas the thermal efficiency is maximized asymptotically when ∆*T_W_* = 0 as follows:

(13) $$\underset{\Delta T_{W}\to 0}{\lim}\eta_{th}=\underset{\Delta T_{W}\to 0}{\lim}\left(1-\frac{T_{C,O}}{T_{W,O}}\right)=\underset{\Delta T_{W}\to 0}{\lim}\left(1-\frac{T_{C}+\Delta T_{C}}{T_{W}-\Delta T_{W}}\right)$$

Now, when $T_{W}\gg \Delta T_{W}$ and $\Delta T_{W}\to 0$ equals $\Delta T_{C}\to 0$, then Equation (13) approaches the maximum thermal efficiency, which is expressed as:

(14) $$\eta_{th,m}=1-\frac{T_{C}}{T_{W}}$$

The discrepancy between the maximum work conditions and the maximum thermal efficiency conditions means that the thermal efficiency is inherently an inconsistent performance evaluation index. Although the thermal efficiency represents the energy conversion efficiency, the heat source for OTEC has a finite temperature and finite flow rate and the heat engine workable temperature range depends on the available heat from heat sources, which is why OTEC heat engines are discussed in FTT. Although the available seawater flow rate is abundant, without the seawater temperature change, the heat will never transfer between the engine and the heat source. The pumping power for the seawater to flow to an OTEC heat engine exceeds the work from the heat engine when drawing much seawater to make the temperature change negligible. Therefore, the temperature change is non-negligible in the OTEC and the flow rate of the heat source can be infinite. The evaluation of the OTEC systems using conventional thermal efficiency as defined in Equation (4) does not elucidate the efficiency of the energy conversion system in OTEC. Evaluating, comparing, and improving the OTEC system requires normalizing the thermal efficiency.

### 2.2. Normalization of the Thermal Efficiency

Understanding the characteristics of the heat source helps recognize the available energy considering the finite temperature and finite flow rate. The total transferable thermal energy between surface seawater and deep seawater must be realized as available heat. The ideal heat transfer process between two heat sources without pressure-drop irreversibility and energy extraction by a heat engine—i.e., the enthalpy difference between heat sources—is given by:

(15) $$H_{W,d}=\int_{T_{0}}^{T_{W}}rC_{HS}dT=rC_{HS}\left(T_{W}-T_{0}\right)=Q_{W,d}$$

(16) $$H_{C,d}=\int_{T_{C}}^{T_{0}}\left(1-r\right)C_{HS}dT=\left(1-r\right)C_{HS}\left(T_{0}-T_{C}\right)=Q_{C,d}$$

where the specific heat of the seawaters are assumed constant during heat transfer and *T*_0_ is the equilibrium temperature [K]; the so-called “dead state” of OTEC where both heat sources finally reach the equilibrium temperature. *Q_W_*_,*d*_ and *Q_C_*_,*d*_ are the heat transfer rate of the heat sources. The heat capacity rate of the heat source *C_HS_* and the heat capacity rate ratio *r* are respectively defined as:

(17) $$C_{HS}={\left(mc_{p}\right)}_{W}+{\left(mc_{p}\right)}_{C}=C_{W}+C_{C}$$

(18) $$r=\frac{\;C_{W}}{C_{W}+C_{C}}=\frac{\;C_{W}}{C_{HS}}$$

(19) $$C_{W}={\left(mc_{p}\right)}_{W}=rC_{HS},\;\;\;\;C_{C}={\left(mc_{p}\right)}_{C}=\left(1-r\right)C_{HS}$$

If there are only heat transfer processes without extracting work from the heat source then *Q_W_*_,*d*_ = *Q_C,d_*, hence:

(20) $$T_{0}=rT_{W}+\left(1-r\right)T_{C}$$

From Equations (12), (13) and (16), a heat source’s transferable thermal energy is:

(21) $$Q_{W,d}=Q_{C,d}=r\left(1-r\right)C_{HS}\left(T_{W}-T_{C}\right)$$

From Equation (21), the enthalpy difference without work extraction for OTEC is the function of the seawater flow rate and the temperature difference between heat sources. Using Equations (17)–(19), Equation (9) can be expressed as:

(22) $$W_{m}=r\left(1-r\right)C_{HS}{\left(\sqrt{T_{W}}-\sqrt{T_{C}}\right)}^{2}$$

According to Equations (21) and (22), *Q_W_*_,*d*_ and *W_m_* are obviously maximized when *r* = 0.5 with constant *C_HS_*. 

**Figure 4:**
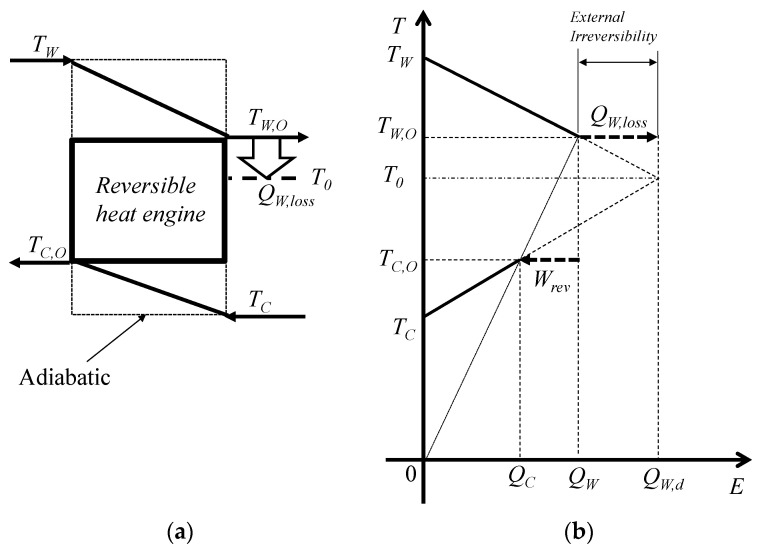
The concept of energy distribution entering the heat engine and the remaining thermal energy of heat sources. (**a**) The conceptual model of the heat engine and heat loss and (**b**) the conceptual *T*–*E* diagram.

The energy balance can be represented by the temperature and energy interaction diagram (*T*–*E* diagram) [37,38,39]. Figure 4 shows the *T*–*E* diagram using a reversible heat engine where the energy conversion losses can be divided into internal and external irreversibility and *Q_W_*_,*loss*_ is the latter. Internal irreversibility indicates the irreversibility of the heat engine. The internal irreversibility is negligible in this study because the reversible heat engine is applied to a heat engine. The external thermal irreversibility is the external energy loss *Q_W_*_,*loss*_ when the heat transfer rate of warm seawater *Q_W_* is subtracted from the transferable thermal energy of the heat source *Q_W_*_,*d*_.

Therefore, considering the external irreversibility of the energy conversion, the heat engine’s normalized thermal efficiency is expressed as:

(23) $$\eta_{th,Nor}=\frac{W}{Q_{W}+Q_{W,loss}}=\frac{W}{Q_{W,d}}$$

For conventional thermal and nuclear power plants, *Q_W_*_,*loss*_ is equivalent to the heat leak in boilers and reactors. In conventional thermodynamics, the efficiency of a heat engine and the heat losses in boilers and reactors can generally be discussed separately because they are never related to the heat engine’s thermal efficiency. However, heat leak is non-negligible for the normalized thermal efficiency of OTEC because the transferable thermal energy is the available energy from the heat source, which is determined by the balance of heat capacity rates between the surface and deep seawater. 

**Figure 5:**
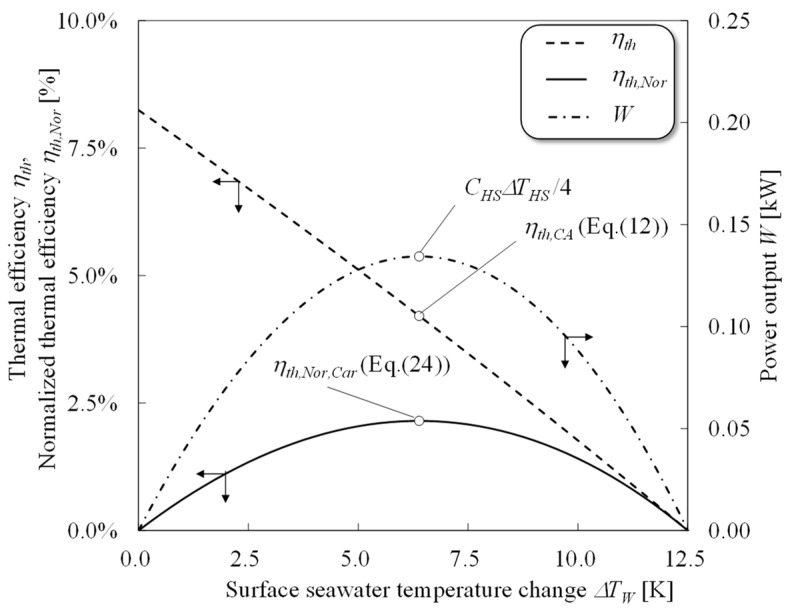
Thermal efficiencies and power output as functions of the surface seawater temperature change in a heat engine. *T_W_* = 303.15 K; *T_C_* = 278.15 K; *C_HS_* = 1 kW/K; *r* = 0.5.

Figure 5 shows the heat engine’s power output, the thermal efficiency, and the normalized thermal efficiency as a function of the surface seawater temperature change. According to Figure 5, conventional thermal efficiency simply decreases as the surface seawater temperature change increases; however, the work from the heat engine and the normalized thermal efficiency will be maximized in the condition of Equations (9)–(11). The conventional thermal efficiency only depends on the available temperature of the heat engine i.e., *T_W_*_,*O*_ and *T_C_*_,*O*_, then the available temperature decreases as the seawater temperature change increases because the conventional thermal efficiency is the ratio between the work from the heat engine and the input thermal energy into the heat engine. Whereas the normalized thermal efficiency is the ratio of the work from the heat engine and the maximum available thermal energy from the heat source, it only depends on the work from the heat engine. Therefore, the maximization of the normalized thermal efficiency corresponds to the increase in the heat engine’s work output, which can effectively show the heat engine’s performance. 

Here, the thermal efficiency of the Carnot heat engine can be derived as:

(24) $$\eta_{th,Nor,Car}=\frac{W_{m}}{Q_{W,d,m}}=\frac{\sqrt{T_{W}}-\sqrt{T_{C}}}{\sqrt{T_{W}}+\sqrt{T_{C}}}=\frac{\Delta T_{HS}}{T_{W}-T_{C}}$$

where $\Delta T_{HS}\equiv {\left(\sqrt{T_{W}}-\sqrt{T_{C}}\right)}^{2}$. When $\sqrt{T_{W}}\gg \sqrt{T_{W}-T_{C}}$, Equation (24) becomes an approximated formula of half of Equation (12). Conventionally, the maximum bound of the Carnot cycle has been well-known as Equation (12) although the thermal efficiency is not its maximum. However, after normalization considering the equilibrium state as a dead state, Equation (24) will be the maximum thermal efficiency.

## 3. Second Law of Thermodynamics

### 3.1. Available Work Maximization 

**Figure 6:**
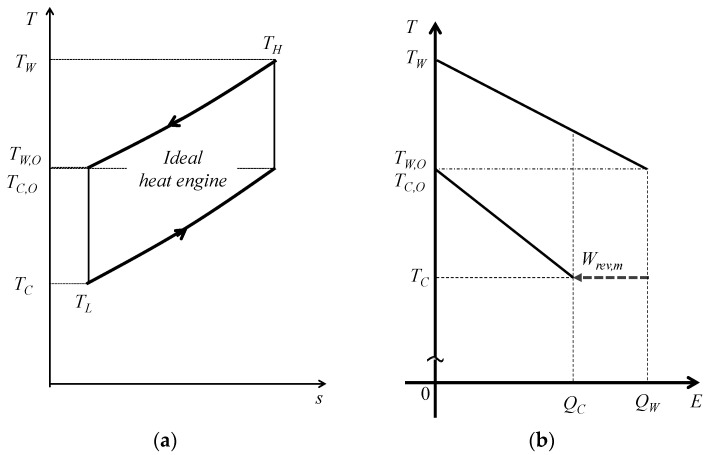
Conceptual diagrams of an ideal reversible heat engine. (**a**) *T*–*s* diagram, and (**b**) *T*–*E* diagram.

The available energy from the heat sources, the so-called “exergy,” is the work output by means of the ideal reversible heat engine, of which the heat engine can ideally be varied. Figure 6 shows the conceptual *T*–*s* and *T*–*E* diagrams of an OTEC using an ideal heat engine with ideal heat transfer. As shown in Figure 6, the heat exchange between the heat source and the heat engine is the counter flow and the working temperature of this ideal heat engine can vary alongside the heat source temperature. Then, the cold heat source temperature starts at *T_C_*_,*O*_ in Figure 6b. Therefore, the difference between the exergy and the actual power output is the loss as the system generates entropy. During the heat transfer process between seawater and the heat engine, the total entropy generation *S_gen_*_,*HE*_ yielded by the temperature change in seawaters can be calculated by:

(25) $$\begin{array}{l} S_{gen,HE}=\Delta S_{W,\Delta T}+\Delta S_{C,\Delta T}+\Delta S_{W,\Delta P}+\Delta S_{C,\Delta P}\\ \;\;\;\;\;\;\;=C_{ws}ln\frac{T_{W}}{T_{W,O}}+C_{cs}ln\frac{T_{C,O}}{T_{C}}+\Delta S_{W,\Delta P}+\Delta S_{C,\Delta P}\\ \;\;\;\;\;\;\;=rC_{HS}ln\frac{T_{W}}{T_{W,O}}+\left(1-r\right)C_{HS}ln\frac{T_{C,O}}{T_{C}}+\Delta S_{W,\Delta P}+\Delta S_{C,\Delta P} \end{array}$$

where the subscription of ∆*T* and ∆*P* describe a temperature change and pressure drop, respectively. For OTEC, the pressure drop depends on the operating condition, the piping specifications, the heat exchanger, the construction site, etc. In addition, the ideal heat exchanger for which the entropy generated by the pressure drop can be ignored is taken into consideration for the exergy, although seawater pumping powers must not be neglected in relation to the net power output throughout the entire system. In an ideal heat exchange process, the entropy generation would be zero, hence:

(26) $$T_{W,O}=T_{W}{\left(\frac{T_{C}}{T_{C,O}}\right)}^{\frac{1-r}{r}}$$

(27) $$T_{C,O}=T_{C}{\left(\frac{T_{W,O}}{T_{W}}\right)}^{\frac{1-r}{r}}$$

Then, the available work output using the ideal reversible heat engine *W_rev_* will be one degree of freedom of *T_W_*_,*O*_ or *T_C_*_,*O*_ and the maximum available work output can be achieved by $\partial W_{rev,m}/\partial T_{W,O}=0$ or $\partial W_{rev,m}/\partial T_{C,O}=0$. Finally, the maximum available work output *a* and the optimum outlet temperature of warm and cold seawaters *T_W_*_,*O*,*opt*_, *T_C_*_,*O*,*opt*_ will be:

(28) $$W_{rev,m}=C_{HS}T_{W}\left[r+\left(1-r\right)\left(\frac{T_{C}}{T_{W}}\right)-{\left(\frac{T_{C}}{T_{W}}\right)}^{1-r}\right]$$

(29) $$T_{W,O,opt}=T_{C,O,opt}=T_{W}{\left(\frac{T_{C}}{T_{W}}\right)}^{1-r}$$

The optimum outlet temperatures *T_W_*_,*O*,*opt*_ and *T_C_*_,*O*,*opt*_ will be the same temperature so the heat source will reach an equilibrium state after energy conversion.

### 3.2. Exergy and Entropy Generation

The difference between the enthalpy and entropy gaps in the dead state define the exergy *E_x_*:

(30) $$E_{x}=\Delta H-T_{0}\Delta S=\left(H-H_{0}\right)-T_{0}\left(S-S_{0}\right)$$

Applying the above equation to both heat sources:

(31) $$\begin{array}{l} E_{x}=Q_{W,d}+Q_{C,d}-rC_{HS}T_{0}\int_{T_{0}}^{T_{W}}\frac{dT}{T}-\left(1-r\right)C_{HS}T_{C}\int_{T_{C}}^{T_{0}}\frac{dT}{T}\\ \;\;\;\;\;\;\;=2r\left(1-r\right)C_{HS}\left(T_{W}-T_{C}\right)\\ \;\;\;\;\;\;\;-C_{HS}\left[rT_{0}ln\left(\frac{T_{W}}{T_{0}}\right)+\left(1-r\right)T_{C}ln\left(\frac{T_{0}}{T_{C}}\right)\right] \end{array}$$

Here, the dead state *T*_0_ is defined as Equation (20) rather than the atmospheric temperature because the system is considered to have a finite quantity of heat sources and an adiabatic system as shown in Figure 1. Equations (28) and (31) are both the maximized work from the heat source thermal energy.

**Figure 7:**
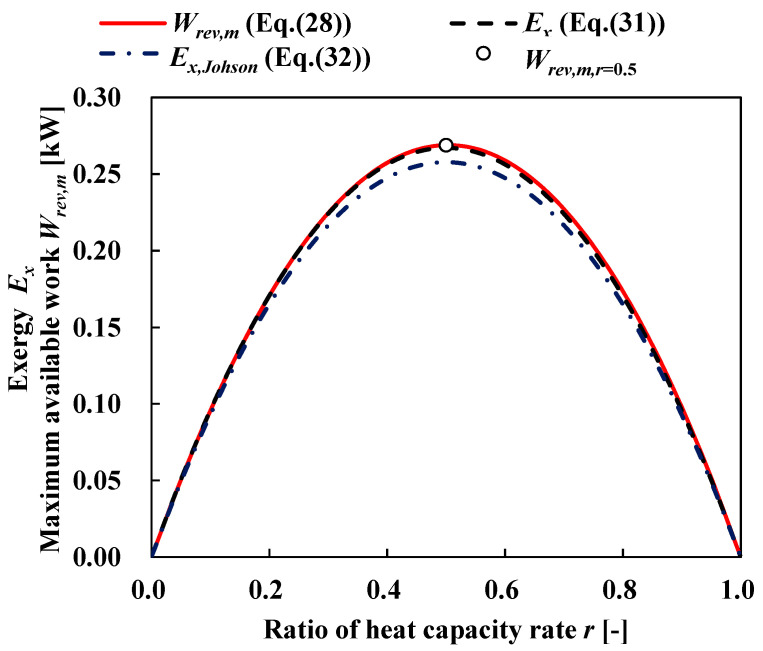
Exergy and maximum available work as a function of heat capacity rate ratio. *T_W_* = 303.15 K; *T_C_* = 278.15 K; *C_HS_* = 1 kW/K.

Figure 7 shows the relationship between the maximum available work *W_rev_*_,*m*_ as defined by Equations (28) and (31) as a function of the ratio of the heat source’s heat capacity rate. Figure 7 also shows the exergy as defined by Johnson [40] as follows:

(32) $$E_{x,Johson}=\frac{C_{HS}T_{W}}{8}\eta_{th,m}{}^{2}=\frac{C_{HS}T_{W}}{8}{\left(1-\frac{T_{C}}{T_{W}}\right)}^{2}$$

According to Figure 7, the maximum available work and Equation (28) correspond to the entire ratio of the heat capacity rate. Therefore, *W_rev_*_,*m*_ is the exergy of the thermal energy *Ex_HS_* in the ocean for OTEC. Here, the exergy efficiency *η_th_*_,*Ex*_ is defined as:

(33) $$\eta_{th,Ex}=\frac{W}{Ex_{HS}}=\frac{W}{C_{HS}T_{W}\left[r+\left(1-r\right)\left(\frac{T_{C}}{T_{W}}\right)-{\left(\frac{T_{C}}{T_{W}}\right)}^{1-r}\right]}$$

Then, the exergy efficiency of the Carnot cycle is calculated as:

(34) $$\eta_{th,Ex,Car}=\frac{W_{m}}{Ex_{HS}}=\frac{r\left(1-r\right)\Delta T_{HS}}{T_{W}\left[r+\left(1-r\right)\left(\frac{T_{C}}{T_{W}}\right)-{\left(\frac{T_{C}}{T_{W}}\right)}^{1-r}\right]}$$

where $\Delta T_{HS}\equiv {\left(\sqrt{T_{W}}-\sqrt{T_{C}}\right)}^{2}$.

Considering the external heat loss to the dead state, entropy generation in the process of thermal energy conversion for the heat source is defined as [19,41]:

(35) $$S_{gen,\;HS}=\frac{Q_{loss,W}+Q_{C}}{T_{C}}$$

**Figure 8:**
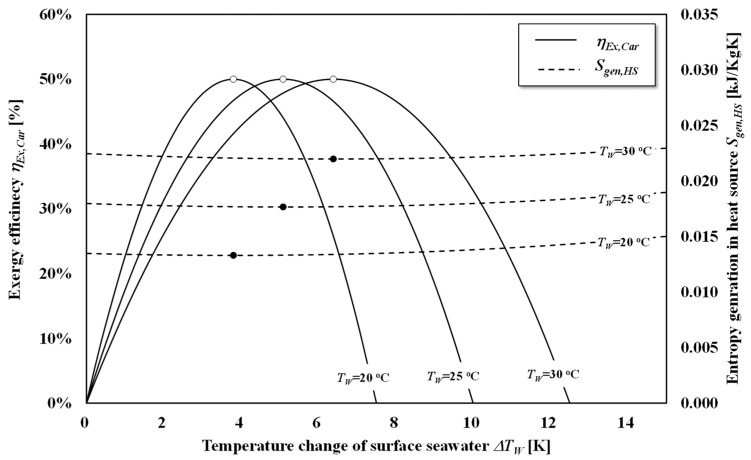
Relationship between the exergy efficiencies and entropy generation in heat sources as a function of the temperature change in surface seawater. *T_C_* = 278.15 K; *C_HS_* = 1 kW/K; *r* = 0.5. The open circles show the maximum points and closed circles show the minimum points.

Figure 8 shows the relationship between the exergy efficiency and entropy generation in the process of the Carnot heat engine shown in Figure 4. According to Figure 8, entropy generation is minimized at the maximum work without considering the external heat loss *Q_W_*_,*loss*_ based on the equilibrium state and, thus, the results of entropy generation minimization will show the discrepancy due to the accompaniment of a dead state as the atmospheric temperature. However, the results in Figure 8 show that minimizing the entropy generation is equivalent to maximizing the system’s power output by introducing the equilibrium temperature state as the dead state.

### 3.3. Ideal and Staging Carnot Heat Engines 

Morisaki and Ikegami proposed the theoretical maximum work from the staging Carnot cycle [31]. The N-stage Carnot cycle maximum work output *W_Car_*_,*m*,*N*_, optimum warm seawater heat transfer rate *Q_W_*_,*opt*,*N*_, and optimum cold seawater heat transfer rate *Q_C_*_,*opt*,*N*_ can be theoretically formulated as follows:

(36) $$W_{Car,m,N}=Q_{W,opt,N}-Q_{C,opt,N}$$

(37) $$Q_{W,opt,N}=rC_{HS}\left[T_{W}-{\left\{r\;\;T_{W}{}^{\frac{1}{N}}+\left(1-r\right)\;T_{W}{}^{\frac{1}{N\left(N+1\right)}}\;T_{C}{}^{\frac{1}{N+1}}\right\}}^{N}\right]$$

(38) $$Q_{C,opt,N}=\left(1-r\right)C_{HS}\left[{\left\{\left(1-r\right)T_{C}{}^{\frac{1}{N}}+r\;T_{C}{}^{\frac{1}{N\left(N+1\right)}}T_{W}{}^{\frac{1}{N+1}}\;\;\right\}}^{N}-T_{C}\right]$$

Here, the optimum outlet temperature of warm surface seawater and cold deep seawater *T_W_*_,*opt*,*N*_ and *T_C_*_,*opt*,*N*_ can be calculated as follows:

(39) $$T_{W,opt,N}={\left\{r\;\;T_{W}{}^{\frac{1}{N}}+\left(1-r\right)\;T_{W}{}^{\frac{1}{N\left(N+1\right)}}\;T_{C}{}^{\frac{1}{N+1}}\right\}}^{N}$$

(40) $$T_{C,opt,N}={\left\{r\;\;T_{W}{}^{\frac{1}{N+1}}\;T_{C}{}^{\frac{1}{N\left(N+1\right)}}+\left(1-r\right)\;T_{C}{}^{\frac{1}{N}}\right\}}^{N}$$

**Figure 9:**
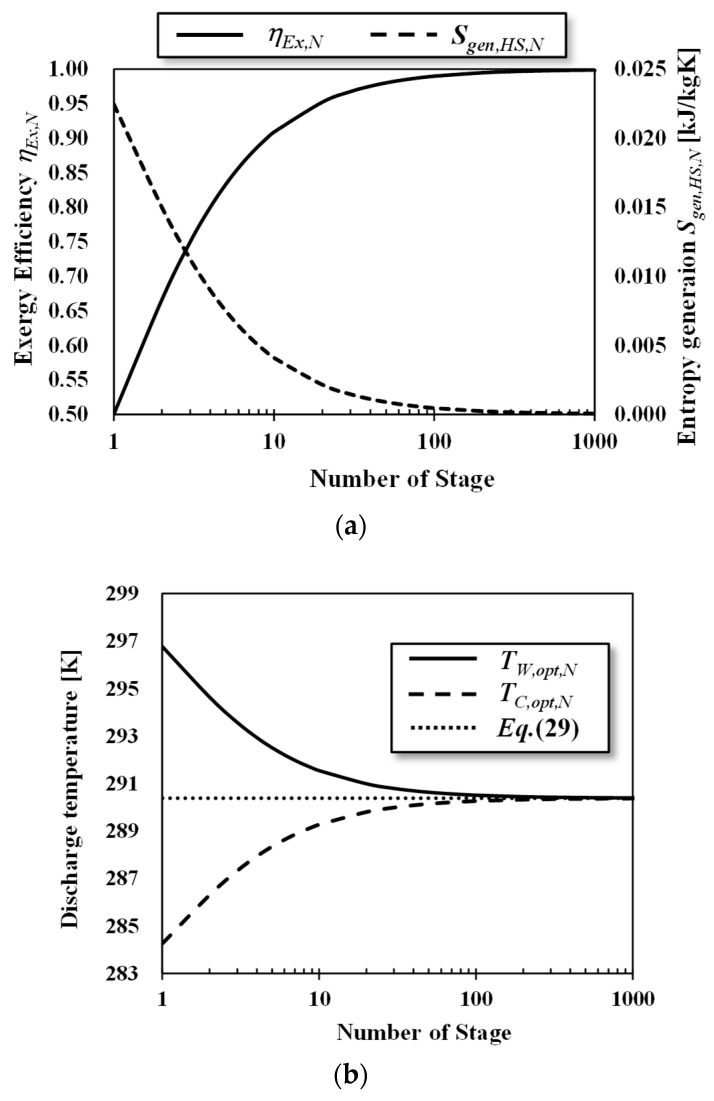
The staging Carnot cycle (**a**) exergy efficiency and entropy generation of heat source, and (**b**) heat-source discharge temperatures as functions of the number of stages. *T_W_* = 303.15 K; *T_C_* = 278.15 K; *C_HS_* = 1 kW/K; *r* = 0.5.

Figure 9 shows (a) the exergy efficiency and entropy generation of a heat source, and (b) the discharge temperature as a function of the number of stages. According to Figure 9a, the exergy efficiency of the staging Carnot cycle approaches 1.0 as the number of stages increases and the entropy generation decreases as the number of stages increases. According to Figure 9b, the discharge temperature of warm surface seawater in each stage *T_W_*_,*O*,*opt*,*N*_ and *T_C_*_,*O*,*opt*,*N*_ form an asymptotic curve in relation to the dead state temperature as expressed in Equation (29). As this temperature approaches an increase in the number of stages, the *Q_loss_*_,*W*_ and *S_gen_*_,*HS*_ decrease. This is because the effective temperature of staging heat engine will increase, although the temperature difference between the outlet of heat sources decrease as the stage increases. The optimized staging is the series of the heat engine that utilizes heat effectively and the thermal efficiency of the total staging heat engine corresponds to Equation (12).

For the practical design, considering the increased quantity and cost requirement, the number of stages will be three because of the small heat source temperature difference [7,8]. The exergy efficiency of double-stage and triple-stage Carnot cycles are 0.67 and 0.75, which correspond to 33% and 50% increases from the single-stage, respectively. 

## 4. Performance Evaluation of OTEC Heat Engines

In the OTEC system, the practical maximum temperature range between the warm surface seawater and cold deep seawater will be 288.15–303.15 K and the work produced by the ideal heat engine (*W_rev_*_,*m*_) will be maximized when *r* = 0.502–0.504. In general, the flow rate balance allows free design in an OTEC system; therefore, the potential of OTEC will be $E_{x,HS,r=0.5}=\frac{C_{HS}}{2}\Delta T_{HS}$. Then, the upper bound of the available work for a simple Carnot heat engine is half of the exergy.

One theoretically realizable reversible heat engine type is an infinite multi-stage Carnot cycle; Figure 6 shows the conceptual *T*–*s* diagram for this. The normalized thermal efficiency of an ideal heat engine can be expressed as:

(41) $$\eta_{th,Nor,m}=\frac{W_{rev,m}}{Q_{W,d}}=\frac{r+\left(1-r\right)\left(\frac{T_{C}}{T_{W}}\right)-{\left(\frac{T_{C}}{T_{W}}\right)}^{1-r}}{r\left(1-r\right)\left[1-\left(\frac{T_{C}}{T_{W}}\right)\right]}$$

The formula can be calculated for *r* = 0.5:

(42) $$\eta_{th,\;Nor,m}=2\frac{\sqrt{T_{W}}-\sqrt{T_{C}}}{\sqrt{T_{W}}+\sqrt{T_{C}}}=\frac{2\Delta T_{HS}}{T_{W}-T_{C}}$$

When $\sqrt{T_{W}}\gg \sqrt{T_{W}-T_{C}}$, Equation (42) approximately corresponds to Equation (12).

Table 1 summarizes the maximum works of ideal cycles, performance evaluation methods, and the potential. Typical practical heat engines for the Carnot cycle and for the ideal cycle can be both the Rankine cycle using pure working fluid and the Claud cycle, or the Uehara cycle [4] and the Kalina cycle [3] that use a non-azeotropic mixture as a working fluid. The potential described in Table 1 shows that *r* will be 0.5 for the design of an OTEC heat engine with the maximum power and minimum seawater consumption. The practical potential of OTEC was proposed by Nihous based on assumptions about the available flow rate of seawater and typical internal energy consumption [42,43]. In consideration of the internal energy consumption to operate the OTEC heat engine, the quantity of entropy generation due to the performance of every component must be calculated, although the model of Nihous assumes that 30% of generated power is consumed as seawater intake power. In the actual case, entropy generation due to the performance of heat exchangers, irreversibility in heat engines, and the pressure drop in piping and heat exchangers has to be taken into account, although our model neglects the losses in the heat exchanger process and in heat engines to focus on the essential concept of the heat engine, i.e., *∆S_W_*_,*∆P*_ and *∆S_C_*_,*∆P*_ in Equation (25) are non-negligible. 

Table 2 shows the theoretically optimized OTEC performance results in a conventional study. The power output shows the power from the heat engine without seawater pumping power. The exergy *Ex_HS_* is calculated in the heat source condition by Equation (28); the maximum work *W_m_*, which depends on the heat engine, is estimated by Equations (22), (28) and (36); the thermal efficiency *η_th_* uses the conventional first law efficiency (Equation (4)); Equation (23) defined the normalized thermal efficiency *η_th_*_,*Nor*_; Equation (33) expresses the exergy efficiency. Case 2 and Case 3 have the same *η_th_*; however, the hex of Case 2 is much higher than Case 3 and the *η_th_* of Case 4 is higher than for Case 1; however, Case 1 has the higher hex. With the same heat source temperature condition, the comparison between Case 1 and Case 5 shows that Case 5 has a much higher hex. If the system applies the higher *W_m_*/*E_xHS_* heat engine, the hex tends to increase accordingly except in Case 9. In Case 9, using the Kalina cycle, even though it has the highest *W_m_*/*E_xHS_*, the hex is smaller than most Rankine cycle cases. The result in Table 2 is estimated under various different assumptions, including the performance of heat exchangers, turbines, and working fluid pump; however, the object function for the optimization must be selected adequately to maximize the efficiency and the energy conversion performance will be expressed by the hex and *η_th_*_,*Nor*_. Although economic analysis is also required in the optimization of the OTEC system design, the proposed evaluation method is effective for evaluating the practical OTEC design and optimization. To evaluate the system performance, the normalized thermal efficiency (Equation (23), and the exergy efficiency (Equation (33)) are applicable for evaluating the system if the *W* in each equation uses a net power. 

## 5. Conclusions

The conventional definition of the thermal efficiency certainly recognizes the system’s efficiency; however, this efficiency only shows the ratio of thermal energy and the work from the heat engine because the maximum work condition and maximum thermal efficiency condition are different. Therefore, this study proposes a model to normalize the thermal efficiency that considers the equilibrium state of the heat source where the transferable thermal energy is added heat and the loss of thermal energy. Based on the model, the maximum condition corresponds to the normalized thermal efficiency maximum points. Moreover, the maximum available energy is proposed by entropy generation minimization and compared to the exergy’s general definition. The model newly proposes the exergy of OTEC and shows that the maximum exergy efficiency condition corresponds to the minimum entropy generation condition. Therefore, the performance evaluation method is definitely useful and applicable in the system design and optimization. For the construction of fundamental thermodynamics, the performance evaluation method is applied to the ideal heat engines to derive the normalized thermal efficiency of the Carnot cycle, revealing that the exergy efficiency of a simple Carnot cycle is 50% and that of an infinite staging Carnot cycle is 100%. In addition, a method that permits the calculation of the theoretical potential of OTEC, and the normalized thermal efficiency (first law) and the exergy efficiency (second law) can be applied to evaluate even a practical OTEC system if the power considers the system’s internal power consumption. Finally, the evaluation method can be applied to the theoretical optimized design condition and shows the applicability and effectiveness in the practical heat engines.

## Figures and Tables

**Table 1:** Summary of maximum work, potential, and efficiencies for performance evaluation.

Ideal Heat Engines	Carnot Cycle	Ideal Cycle
**Practical heat engines**	Rankine and Claud	Uehara and Kalina
**Maximum work**	$r\left(1-r\right)C_{HS}\Delta T_{HS}$	$C_{HS}T_{W}\left[r+\left(1-r\right)\left(\frac{T_{C}}{T_{W}}\right)-{\left(\frac{T_{C}}{T_{W}}\right)}^{1-r}\right]$
**Potential (*r* = 0.5)**	$\frac{C_{HS}\Delta T_{HS}}{4}$	$\frac{C_{HS}\Delta T_{HS}}{2}$
**Normalized thermal efficiency**	$\frac{\Delta T_{HS}}{T_{W}-T_{C}}$	$\frac{r+\left(1-r\right)\left(\frac{T_{C}}{T_{W}}\right)-{\left(\frac{T_{C}}{T_{W}}\right)}^{1-r}}{r\left(1-r\right)\left[1-\left(\frac{T_{C}}{T_{W}}\right)\right]}$
**Exergy efficiency**	$\frac{r\left(1-r\right)\Delta T_{HS}}{T_{W}\left[r+\left(1-r\right)\left(\frac{T_{C}}{T_{W}}\right)-{\left(\frac{T_{C}}{T_{W}}\right)}^{1-r}\right]}$	1

**Table 2:** Comparison of the theoretical optimum designs of OTEC.

Case	1	2	3	4	5	6	7	8	9	10
Heat engine (Stages)	Rankine	Rankine	Rankine	Rankine	Rankine	Rankine (Two)	Rankine (Two)	Rankine (Three)	Kalina	Kalina
*W* (kW)	3877	5750	15,733	20	5000	6350	6079	6425	20	6420
*T_W_* (°C)	28	29	28	26	28	29	28	28	26	29
*T_C_* (°C)	4	6	5	5	4	6	4	4	5	6
*C_HS_* (kW/K)	69192	69856	347476	505	66891	69856	66891	66891	505	69856
*r*	0.51	0.50	0.59	0.51	0.51	0.50	0.51	0.51	0.51	0.50
*W_m_*/*E_xHS_*	50%	50%	50%	50%	50%	67%	67%	75%	91%	90%
*η_th_*	2.2%	3.2%	3.2%	2.4%	3.1%	3.2%	3.7%	3.9%	2.4%	3.2%
*η_th_*_,*Nor*_	0.93%	1.43%	0.81%	0.76%	1.25%	1.58%	1.52%	1.60%	0.76%	1.60%
*η_EX_*	22.5%	36.2%	20.4%	20.7%	30.0%	39.9%	36.5%	38.6%	20.7%	40.4%
Reference	[44]	[7]	[11]	[45]	[8]	[7]	[8]	[8]	[45]	[7]

## Nomenclature

*C*	(kW/K)	Heat capacity
*c_p_*	(kJ/(kg K))	Specific heat
*Ex*	(kW)	Exergy
*H*	(kJ)	Enthalpy
*m*	(kg/s)	Mass flow rate
*Q*	(kW)	Heat flow rate
*R*	(-)	Ratio of warm seawater heat-source Heat capacity
*S*	(kJ/(kg K))	Entropy
*T*	(K)	Temperature
*ΔT*	(K)	Temperature difference
*ΔT_HS_*	(K)	${\left(\sqrt{T_{W}}-\sqrt{T_{C}}\right)}^{2}$
Greek Symbols		
*η*	(%)	Efficiency
Subscripts	
*C*	Cold deep seawater
*CA*	Curzon-Ahlborn
*Car*	Carnot cycle
*d*	Transferable
*H*	High-temperature working fluid temperature on heat engine
*HS*	Heat source
*L*	Low-temperature working fluid temperature on heat engine
*loss*	Lost thermal energy (defines as the difference from the equilibrium state)
*N*	Number of stage
*Nor*	Normalized
*m*	Maximized
*O*	Outlet
*opt*	Optimum
*rev*	Reversible
*th*	Thermal
*W*	Warm surface seawater
